# Analytical Modeling and Analysis of Permanent-Magnet Motor with Demagnetization Fault

**DOI:** 10.3390/s22239440

**Published:** 2022-12-02

**Authors:** Cenwei Shi, Lin Peng, Zhen Zhang, Tingna Shi

**Affiliations:** College of Electrical Engineering, Zhejiang University, Hangzhou 310027, China

**Keywords:** permanent-magnet motor, demagnetization fault, analytical modeling

## Abstract

Factors such as insufficient heat dissipation and excessively high temperature can easily lead to demagnetization of the PMs in permanent-magnet (PM) motors. As a result, the magnetic field distribution of the motor will not be uniform, producing fault harmonics and lowering the operational performance of the motor. An essential stage in the diagnosis of faults and the monitoring of motor condition is the establishment of an accurate model of motors with demagnetization faults. In this paper, demagnetization faults are modeled by changing the Fourier coefficients in the Fourier expansion of the magnetization of PMs. This model can be used to determine the motor performance under various types of demagnetization, including radial air gap flux density, back electromotive force (EMF), and torque. On this basis, the corresponding relationship between the demagnetization degree and the fault signature is established, to provide a theoretical foundation for the subsequent demagnetization fault diagnosis. The finite element analysis (FEA) verifies the effectiveness and superiority of the proposed analytical model. The modeling method proposed in this paper can be applied to PM motors with PMs having different magnetization directions and shapes because it is based on the demagnetization region of PMs.

## 1. Introduction

PM motors have a broad application prospect in the industrial field, profiting from the advantages of high power density and high efficiency [1]. As the source of power, if the PM motor is subjected to faults, it will impact the operating performance or even threaten the operation safety of the motor [2]. The common faults of PM motors include demagnetization faults, short-circuit faults, and eccentricity faults. In application scenarios such as electrical vehicles and aerospace, the heat dissipation conditions are usually limited. As a result, the PM motors’ high power density will lead to the high operating temperature of the motors, and the knee point of PM demagnetization curve rises. When the operating point moves below the knee point, irreversible demagnetization fault will occur. In addition, cooling system malfunctions, short-circuit faults, and aging of magnets can also lead to demagnetization faults. After the demagnetization fault occurs, the stator current increases in order to generate enough torque. This will lead to an increase in copper consumption and heat, which further aggravates the demagnetization fault and forms a vicious cycle [3,4]. Therefore, early demagnetization fault diagnosis is very critical to prevent the further deterioration of motor performance and minimize possible losses. At present, there are three commonly used demagnetization fault diagnosis methods: the signal-based fault diagnosis methods, the knowledge-based fault diagnosis methods, and the model-based fault diagnosis methods [5].

(1) The signal-based fault diagnosis method extracts the fault characteristics from the measured signals such as voltage [6,7,8], current [6,7,9,10], flux [11,12], and torque [13] of the motor for fault diagnosis using signal processing techniques. In [6], harmonic analysis was carried out on signals such as no-load back-EMF, line current, and the zero-sequence voltage component of the motor with demagnetization faults, and the differences in the harmonic content of various signals between the healthy motor and the partially demagnetized motor were calculated; this served as the basis for diagnosing demagnetization faults. In [11], rotor eccentricity fault and partial demagnetization fault were diagnosed using the directly measured magnetic flux inside the motor. This method does not require the exact internal structure of the motor. It just requires using the differences in signals between the health state of the motor and the fault state to extract the fault characteristics and achieve fault diagnosis. It has been widely used in the field of fault diagnosis. However, this method needs to know the fault characteristic signal and its frequency in advance, and the diagnostic accuracy may be impacted by operating conditions (load and speed).

(2) The knowledge-based fault diagnosis method trains the fault diagnosis capability of artificial intelligence through a large number of fault operation data to realize the fault diagnosis and classification in complex cases in engineering applications. The expert system [14], neural network [15], support vector machine [16], and deep learning [17,18,19,20,21] are examples of frequently used intelligent diagnosis methods. In [17], a deep-learning-based stacked auto-encoder technology was implemented, which used current and vibration data to identify various faults of motors under different operating conditions. In [18], a one-dimensional convolutional neural network model was established. It extracted the fusion features of motor current and torque signals through multiple convolutional feature-extraction modules to realize the classification of demagnetization faults and bearing faults as well as the diagnosis of eccentricity. In [20], a new unsupervised fault diagnosis model was developed. It successfully extracted the deep coding features that can represent the original input and achieved directional node information fusion to construct deep representation features. This method does not need the establishment of a mathematical model for the motor or the provision of a prior accurate analysis, which can eliminate the interference of human factors in fault characteristic extraction. It offers a wide range of potential applications, including fault detection and diagnosis in complex systems. However, this method relies on the quantity and quality of fault operation data to train the ability of fault diagnosis of artificial intelligence. Hence, it is limited in practical applications. In addition, the fault features with actual physical significance are not available using this method, and the long computing time makes it difficult to apply to real-time diagnosis.

(3) The model-based fault diagnosis method constructs the mathematical model of the motor to estimate the operating states or the performance parameters of the motor. Based on this mathematical model, the performance parameters of the motor under different operating conditions can be calculated. The fault diagnosis is realized by monitoring the difference between the actual performance and the predicted performance of the motor. This method is not limited by operating conditions, nor does it require a large amount of fault operation data or a complex signal processing procedure. It has the advantages of non-invasion and low cost, but it needs an accurate model of the motor, and the effectiveness of the diagnosis is highly dependent on the accuracy of the model. The model-based fault diagnosis methods are mainly divided into the numerical method, the magnetic equivalent circuit method, and the analytical method.

The finite element method, which is the most popular numerical method, simplifies the mathematical problem that needs to be solved into a series of arithmetic operations and logic operations by meshing the solving region of the motor and listing the approximate linear algebraic equations. A variety of finite element calculation software, such as Ansys, continues to develop as a result of the development of computers. The demagnetization fault model of a motor is generally established by changing the remanence or magnetic coercivity of the materials of the PM [22,23] or the volume of the PM [9] in software. It has the advantages of mature application and high calculation accuracy, but it demands a lot of computing time and resources, and the number of elements will also affect the accuracy of the results. Moreover, this method cannot directly reflect the physical relationship between the motor’s performance and parameters.

In the magnetic equivalent circuit method, the actual non-uniformly distributed magnetic field is regarded as a multi-section average magnetic circuit, and then the calculation is carried out by analogy with the calculation criteria in the electric circuit. The demagnetization fault model of the motor is generally established by changing the magnetomotive force [22,23] or equivalent magnetic flux and reluctance [24] of the demagnetized PM in the magnetic circuit model. In [22], by changing the equivalent magnetomotive force of the demagnetized PM, a mathematical model of the tooth flux of the stator teeth within the range of demagnetized magnetic poles can be established to diagnose the number of demagnetized poles and the degree of demagnetization. In [24], the leakage flux outside the generator with a demagnetization fault was calculated by adjusting the equivalent flux and reluctance corresponding to the demagnetized PM, which was utilized as the basis for fault diagnosis. The magnetic equivalent circuit method has the advantages of a clear description of the motor magnetic circuit model and simple calculation. However, this method assumes that the magnetic flux is distributed evenly along the section and length of each magnetic circuit. As a result, some local structural characteristics of the motor are difficult to reflect, and only the average value with low precision in several areas can be obtained. In addition, some parameters of the motor such as flux leakage coefficient and pole-arc coefficient are difficult to calculate using this method.

The analytical method is based on the electromagnetic field equations of different regions of the motor and their boundary conditions to establish the analytical model of the motor. It has the advantages of clear physical relationships between various parameters and fast calculation speed, which can be used for real-time health status monitoring. The key to analyzing the demagnetization fault of the motor by analytical method is to establish the model of the demagnetized PM, to define the expression of motor performance parameters. The equivalent current method and the equivalent remanence method are now the two most commonly used methods. The equivalent current method simulates demagnetization faults by transferring partial demagnetization to equivalent current at the sides of the fault PM region. The demagnetization model of the PM can be established by adding one or several pairs of demagnetization-equivalent current to the PM-equivalent current [25,26]. This method is very suitable for modeling partial demagnetization, but the solution procedure is complicated and cannot directly represent the magnetization model of the PM. The equivalent remanence method simulates demagnetization by directly decreasing the remanence or the overall magnetization amplitude of the demagnetized PM. For example, the magnetization of each separate PM was multiplied by a “magnetization factor” to model the partial demagnetization of the motor with radially magnetized magnets [27,28,29]. This method has the advantages of parameterization and fast calculation speed, but it is only applicable to PMs with radial magnetization, and the partial demagnetization of PM is equivalent to the total flux reduction, which cannot simulate the demagnetization of a specific section of PM. Due to the difficulty in modeling, there is little literature on the demagnetization model for parallel magnetized fan-shaped PMs, which is one of the most widely used in practical applications.

The main contributions of this paper include the following: (1) A novel strategy for the demagnetization fault modeling of PM motors is proposed. By changing the waveform of magnetization in the demagnetization region of the PM, the Fourier coefficients in the Fourier expansion of the entire waveform are altered to simulate the uniform demagnetization and the partial demagnetization of a specific region of PM. Compared with previous research, it is closer to the practical situation and has the advantages of intuitive model and clear physical concept. (2) The proposed demagnetization fault analytical model is also effective for parallel magnetized PMs compared with earlier studies. This modeling method based on the demagnetization region is also applicable to PMs with different magnetization directions, shapes, and demagnetization types. (3) The analytical model proposed in this paper takes little calculation time and has great precision, which can provide an accurate reference for further real-time fault diagnosis, prediction, and maintenance planning.

The remainder of this paper is organized as follows. In Section 2, the models of uniform demagnetization and partial demagnetization of PMs are established by changing the Fourier coefficients in the Fourier expansion of the magnetization waveform of PMs. In Section 3, the finite element (FE) model is used to verify the effectiveness and accuracy of the proposed analytical model, and the variations in air gap flux density, flux linkage, back-EMF, and output torque of the motor with the type and degree of demagnetization of the PM are analyzed. Finally, Section 4 summarizes the conclusions of this work.

## 2. Analytical Model for Permanent Magnet

The analytical model is established based on the following assumptions:

(1) Linear magnet properties;

(2) Infinite-permeable iron materials;

(3) Neglected conductivity and eddy-current effects;

(4) Neglected end effects.

**Figure 1:**
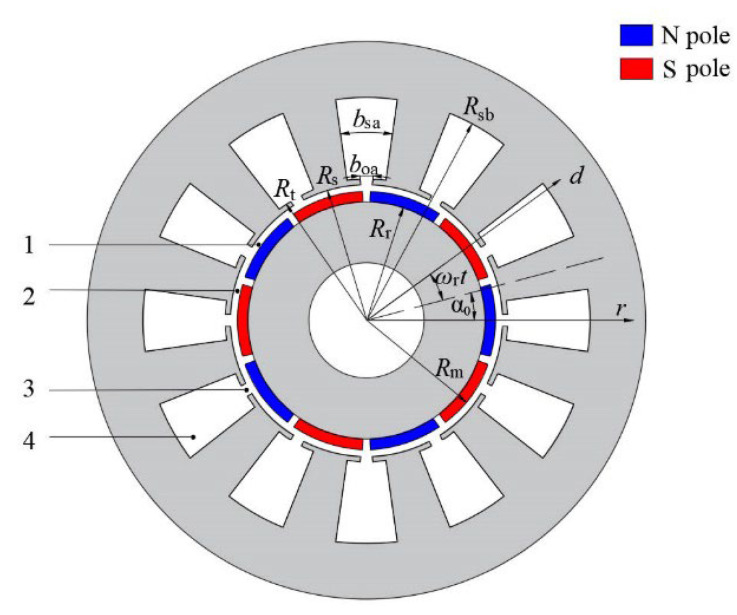
Machine model and four types of subdomains.

The magnetic field solution domain of the motor can be divided into four types of subdomains, as shown in Figure 1, viz., magnet (Region 1), air gap (Region 2), slot opening (Region 3), and slot (Region 4). The corresponding vector potentials are expressed as $A_{z1}$, $A_{z2}$, $A_{z3}$, and $A_{z4}$. $R_{r}$, $R_{m}$, $R_{s}$, $R_{t}$, and $R_{\mathrm{sb}}$ represent the outer radius of the rotor, the outer radius of the magnet, the radius of the stator bore, the radius of the slot top, and the radius of slot bottom, respectively. $b_{\mathrm{oa}}$ and $b_{\mathrm{sa}}$ are the mechanical angles of the slot opening and slot, respectively. $\omega_{r}$ is the rotor rotational speed and $\alpha_{0}$ is the rotor initial position. $\alpha_{t}=\omega_{r}t+\alpha_{0}$, where $\alpha_{t}$ is the angle between the rotor coordinate system and the stator coordinate system at time t.

The following two cases: uniform demagnetization and partial demagnetization, are analyzed. Due to the symmetry, only the demagnetization on the right side of the PM is analyzed. Assume that the demagnetization degree is D (0<D<1) (demagnetization on the left is replaced by 1−D). Here, D=0 represents a healthy PM and D=1 represents total demagnetization.

### 2.1. Uniform Demagnetization

**Figure 2:**
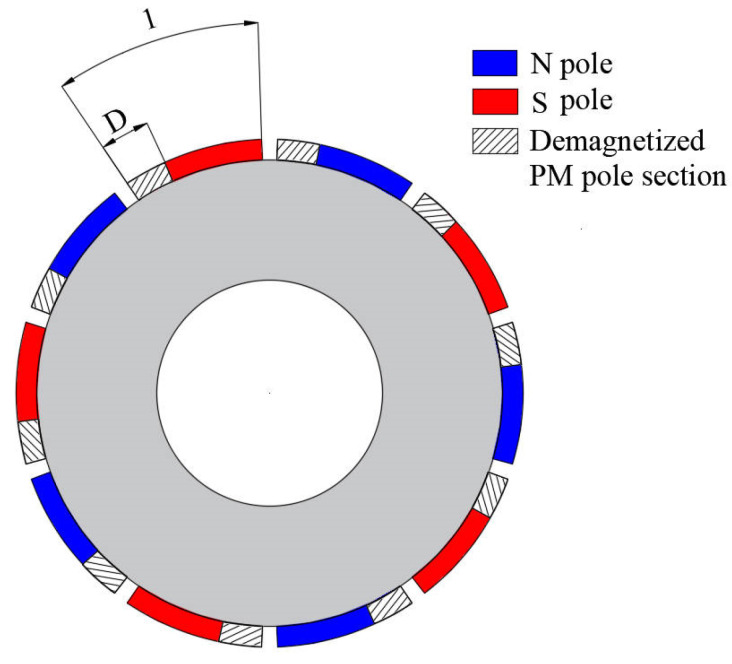
Model of magnets with uniform demagnetization.

In the case of uniform demagnetization, the demagnetization degree of each magnet of the motor is consistent, as shown in Figure 2.

#### 2.1.1. Radial Magnetization

**Figure 3:**
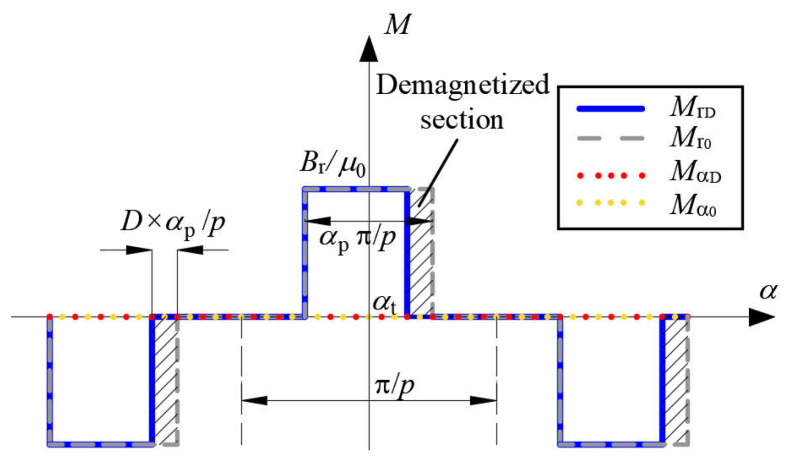
Magnetization of healthy PMs and uniformly demagnetized PMs (radial magnetization).

The radial and tangential components of the magnetization for healthy PMs are *M*_r0_ and $M_{\alpha 0}$, respectively, and for uniformly demagnetized PMs are $M_{\mathrm{rD}}$ and $M_{\alpha D}$, respectively. Their waveforms are shown in Figure 3.

The Fourier decomposition of the radial component of the magnetization of uniformly demagnetized PMs can be given by

(1) $$M_{\mathrm{rD}}=\sum_{k}M_{rk1}\cos(k\alpha -k\alpha_{t})+\sum_{k}M_{rk2}\sin(k\alpha -k\alpha_{t})=\sum_{k}M_{\mathrm{rc}k}\cos k\alpha +M_{\mathrm{rs}k}\sin k\alpha$$

(2) $$\left\{ \begin{array}{l} M_{\mathrm{rc}k}=M_{rk1}\cos(k\alpha_{t})-M_{rk2}\sin(k\alpha_{t})\\ M_{\mathrm{rs}k}=M_{rk1}\sin(k\alpha_{t})+M_{rk2}\cos(k\alpha_{t}) \end{array}\right.$$

for k/p=1,3,5…

(3) $$\{\begin{array}{c} M_{rk1}=\frac{2pB_{r}}{k\pi \mu_{0}}[\sin(\frac{k\pi \alpha_{p}}{2p})+\sin(\frac{k(1-2D)\pi \alpha_{p}}{2p})]\\ M_{rk2}=\frac{2pB_{r}}{k\pi \mu_{0}}[\cos(\frac{k\pi \alpha_{p}}{2p})-\cos(\frac{k(1-2D)\pi \alpha_{p}}{2p})] \end{array}$$

and for k/p≠1,3,5…$M_{rk1}=M_{rk2}=0$.

where p is the number of pole pairs. $B_{r}$ is the remanence of PM. $\mu_{0}$ is the permeability of air. $\alpha_{p}$ is the pole-arc coefficient.

The tangential component of the magnetization of uniformly demagnetized PMs can be expressed by

(4) $$M_{\alpha D}=0$$

#### 2.1.2. Parallel Magnetization

**Figure 4:**
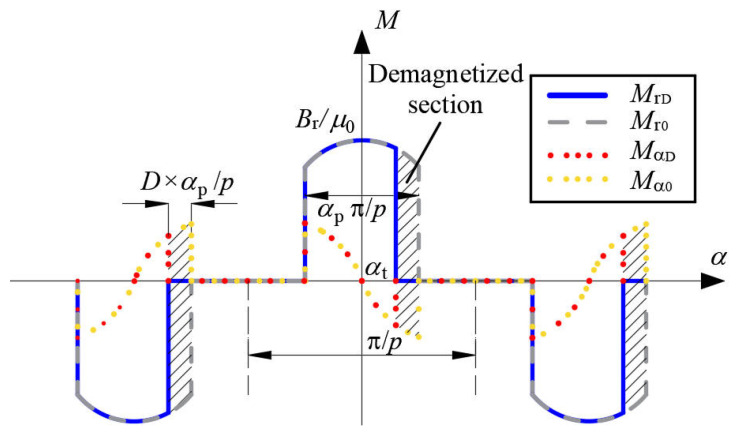
Magnetization of healthy PMs and uniformly demagnetized PMs (parallel magnetization).

The waveforms of radial and tangential components of the magnetization for healthy PMs and uniformly demagnetized PMs are shown in Figure 4.

The Fourier decomposition of the radial component of the magnetization of uniformly demagnetized PMs can be given by

(5) $$\left\{ \begin{array}{l} M_{\mathrm{rD}}=\sum_{k}M_{rk1}\cos(k\alpha -k\alpha_{t})+\sum_{k}M_{rk2}\sin(k\alpha -k\alpha_{t})\\ \;\;=\sum_{k}M_{\mathrm{rc}k}\cos k\alpha +M_{\mathrm{rs}k}\sin k\alpha \\ M_{\alpha D}=\sum_{k}M_{\alpha k1}\cos(k\alpha -k\alpha_{t})+\sum_{k}M_{\alpha k2}\sin(k\alpha -k\alpha_{t})\\ \;\;=\sum_{k}M_{\alpha ck}\cos k\alpha +M_{\alpha sk}\sin k\alpha \end{array}\right.$$

(6) $$\left\{ \begin{array}{l} M_{\mathrm{rc}k}=M_{rk1}\cos(k\alpha_{t})-M_{rk2}\sin(k\alpha_{t})\\ M_{\mathrm{rs}k}=M_{rk1}\sin(k\alpha_{t})+M_{rk2}\cos(k\alpha_{t})\\ M_{\alpha ck}=M_{\alpha k1}\cos(k\alpha_{t})-M_{\alpha k2}\sin(k\alpha_{t})\\ M_{\alpha sk}=M_{\alpha k1}\sin(k\alpha_{t})+M_{\alpha k2}\cos(k\alpha_{t}) \end{array}\right.$$

for k/p=1,3,5…

(7) $$\left\{ \begin{array}{l} M_{rk1}=(S_{1k1}+S_{2k1})B_{r}\alpha_{p}/\mu_{0}\\ M_{rk2}=(S_{1k2}+S_{2k2})B_{r}\alpha_{p}/\mu_{0}\\ M_{\alpha k1}=(-S_{1k2}+S_{2k2})B_{r}\alpha_{p}/\mu_{0}\\ M_{\alpha k2}=(S_{1k1}-S_{2k1})B_{r}\alpha_{p}/\mu_{0} \end{array}\right.$$

where

(8) $$S_{1k1}=\frac{1}{2(k+1)\alpha_{p}\pi /2p}\{\sin\left[(k+1)\alpha_{p}\pi /2p\right]+\sin\left[(k+1)(1-2D)\alpha_{p}\pi /2p\right]\}$$

(9) $$S_{2k1}=\{\begin{array}{ll} \frac{1}{2(k-1)\alpha_{p}\pi /2p}\{\sin\left[(k-1)\alpha_{p}\pi /2p\right]+\sin\left[(k+1)(1-2D)\alpha_{p}\pi /2p\right]\}, & k\neq 1\\ 1-D, & k=1 \end{array}$$

(10) $$S_{1k2}=\frac{1}{2(k+1)\alpha_{p}\pi /2p}\{\cos\left[(k+1)\alpha_{p}\pi /2p\right]-\cos\left[(k+1)(1-2D)\alpha_{p}\pi /2p\right]\}$$

(11) $$S_{2k2}=\{\begin{array}{ll} \frac{1}{2(k-1)\alpha_{p}\pi /2p}\{\cos\left[(k-1)\alpha_{p}\pi /2p\right]-\cos\left[(k-1)(1-2D)\alpha_{p}\pi /2p\right]\}, & k\neq 1\\ 0, & k=1 \end{array}$$

and for k/p≠1,3,5… $M_{rk1}=M_{rk2}=M_{\alpha k1}=$ $M_{\alpha k2}$ =0

### 2.2. Partial Demagnetization

**Figure 5:**
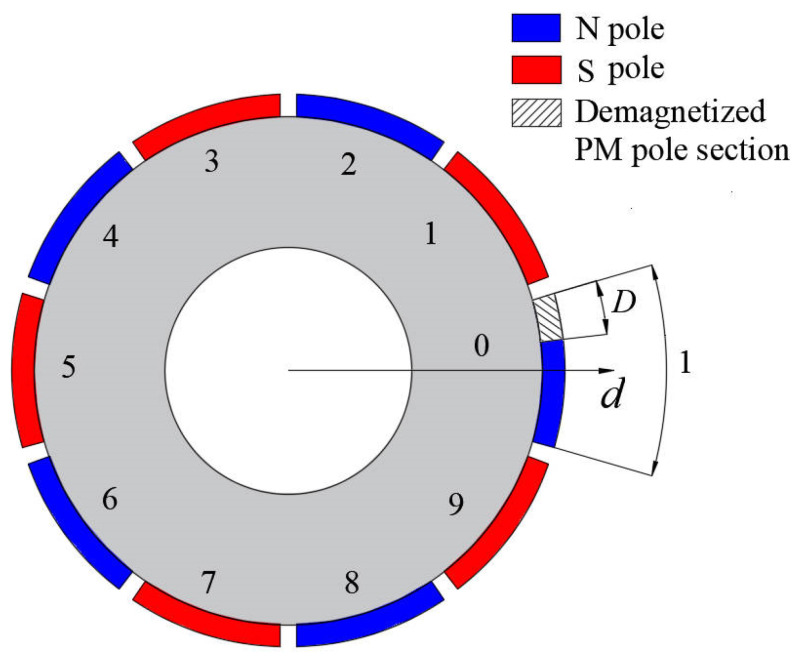
Model of magnets with partial demagnetization.

Assume that i is the number of PMs that are partially demagnetized. i∈{0,1,2,…,2p−1}, as shown in Figure 5.

#### 2.2.1. Radial Magnetization

**Figure 6:**
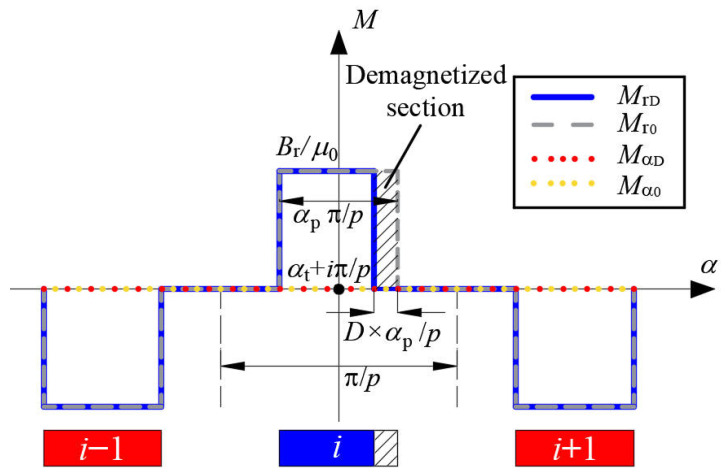
Magnetization of healthy PMs and partially demagnetized PMs (radial magnetization).

The waveforms of radial and tangential components of the magnetization for healthy PMs and partially demagnetized PMs are shown in Figure 6.

The magnetization of partially demagnetized PMs can be viewed as the superposition of the magnetization of the healthy PMs and the effect produced by all the partially demagnetized PMs.

(12) $$\left\{ \begin{array}{l} M_{\mathrm{rD}}=M_{r0}-\sum_{i}M_{\mathrm{rD}i}\\ M_{\alpha D}=M_{\alpha 0}=0 \end{array}\right.$$

where $M_{\mathrm{rD}i}$ is the influence of partial demagnetization of PMs numbered as i on the radial component of magnetization (shaded part). The Fourier decomposition of $M_{\mathrm{rD}i}$ can be given by

(13) $$\begin{array}{l} M_{\mathrm{rD}i}=\frac{a_{0ri}}{2}+\sum_{k}M_{rk1i}\cos(k\alpha -k\alpha_{t})+\sum_{k}M_{rk2i}\sin(k\alpha -k\alpha_{t})\\ \;\;\;=\frac{a_{0ri}}{2}+\sum_{k}(M_{\mathrm{rc}ki}\cos k\alpha +M_{\mathrm{rs}ki}\sin k\alpha ) \end{array}$$

(14) $$\frac{a_{0ri}}{2}={\left(-1\right)}^{i}\frac{B_{r}\alpha_{p}D}{2\mu_{0}p}$$

(15) $$\left\{ \begin{array}{c} M_{\mathrm{rc}ki}=M_{rk1i}\cos(k\alpha_{t})-M_{rk2i}\sin(k\alpha_{t})\\ M_{\mathrm{rs}ki}=M_{rk1i}\sin(k\alpha_{t})+M_{rk2i}\cos(k\alpha_{t}) \end{array}\right.$$

where

(16) $$\left\{ \begin{array}{c} M_{rk1i}={\left(-1\right)}^{i}\frac{B_{r}}{k\mu_{0}\pi }[\sin(\frac{k\pi \alpha_{p}+2ik\pi }{2p})-\sin(\frac{k(1-2D)\pi \alpha_{p}+2ik\pi }{2p})]\\ M_{rk2i}={\left(-1\right)}^{i}\frac{B_{r}}{k\mu_{0}\pi }[-\cos(\frac{k\pi \alpha_{p}+2ik\pi }{2p})+\cos(\frac{k(1-2D)\pi \alpha_{p}+2ik\pi }{2p})] \end{array}\right.$$

The radial component of magnetization of healthy PMs is

(17) $$M_{r0}=\sum_{k}M_{rk0}\cos(k\alpha -k\alpha_{t})=\sum_{k}\left(M_{\mathrm{rc}k0}\cos k\alpha +M_{\mathrm{rs}k0}\sin k\alpha \right)$$

(18) $$\left\{ \begin{array}{c} M_{\mathrm{rc}k0}=M_{rk0}\cos(k\alpha_{t})\\ M_{\mathrm{rs}k0}=M_{rk0}\sin(k\alpha_{t}) \end{array}\right.$$

where

(19) $$M_{rk0}=\{\begin{array}{ll} \frac{4pB_{r}}{k\pi \mu_{0}}\sin\frac{k\pi \alpha_{p}}{2p} & ,\;k/p=1,3,5\ldots \\ 0 & \;,\;k/p\neq 1,3,5\ldots \end{array}$$

According to (13) and (17), (12) can be transformed into

(20) $$\left\{ \begin{array}{l} M_{\mathrm{rD}}=-\sum_{i}\frac{a_{0ri}}{2}+\sum_{k}(M_{\mathrm{rc}k}\cos k\alpha +M_{\mathrm{rs}k}\sin k\alpha )\\ M_{\alpha D}=0 \end{array}\right.$$

where

(21) $$\left\{ \begin{array}{l} M_{\mathrm{rc}k}=M_{\mathrm{rc}k0}-\sum_{i}M_{\mathrm{rc}ki}\\ M_{\mathrm{rs}k}=M_{\mathrm{rs}k0}-\sum_{i}M_{\mathrm{rs}ki} \end{array}\right.$$

#### 2.2.2. Parallel Magnetization

**Figure 7:**
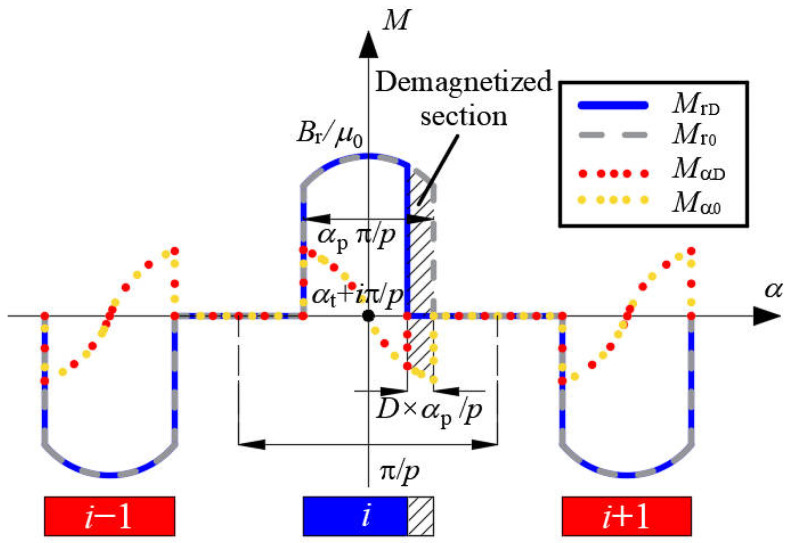
Magnetization of healthy PMs and partially demagnetized PMs (parallel magnetization).

The waveforms of radial and tangential components of the magnetization for healthy PMs and partially demagnetized PMs are shown in Figure 7.

The magnetization of partially demagnetized PMs can be viewed as the superposition of the magnetization of the healthy PMs and the effect produced by all the partially demagnetized PMs.

(22) $$\left\{ \begin{array}{l} M_{\mathrm{rD}}=M_{r0}-\sum_{i}M_{\mathrm{rD}i}\\ M_{\alpha D}=M_{\alpha 0}-\sum_{i}M_{\alpha Di} \end{array}\right.$$

where $M_{\mathrm{rD}i}$ and $M_{\alpha Di}$ are the influence of partial demagnetization of PMs numbered as i on the radial and tangential component of magnetization (shaded part), respectively. The Fourier decomposition of $M_{\mathrm{rD}i}$ and $M_{\alpha Di}$ can be given by

(23) $$\left\{ \begin{array}{l} M_{\mathrm{rD}i}=\frac{a_{0ri}}{2}+\sum_{k}M_{rk1i}\cos(k\alpha -k\alpha_{t})+\sum_{k}M_{rk2i}\sin(k\alpha -k\alpha_{t})\\ \;\;\;\;\;=\frac{a_{0ri}}{2}+\sum_{k}(M_{\mathrm{rc}ki}\cos k\alpha +M_{\mathrm{rs}ki}\sin k\alpha )\\ M_{\alpha Di}=\frac{a_{0\alpha i}}{2}+\sum_{k}M_{\alpha k1i}\cos(k\alpha -k\alpha_{t})+\sum_{k}M_{\alpha k2i}\sin(k\alpha -k\alpha_{t})\\ \;\;\;\;\;=\frac{a_{0\alpha i}}{2}+\sum_{k}(M_{\alpha cki}\cos k\alpha +M_{\alpha ski}\sin k\alpha ) \end{array}\right.$$

(24) $$\left\{ \begin{array}{l} \frac{a_{0ri}}{2}={\left(-1\right)}^{i}\frac{B_{r}}{2\mu_{0}\pi }[\sin(\frac{\alpha_{p}\pi }{2p})-\sin(\frac{\alpha_{p}\pi (1-2D)}{2p})]\\ \frac{a_{0\alpha i}}{2}={\left(-1\right)}^{i}\frac{B_{r}}{2\mu_{0}\pi }[\cos(\frac{\alpha_{p}\pi }{2p})-\cos(\frac{\alpha_{p}\pi (1-2D)}{2p})] \end{array}\right.$$

(25) $$\left\{ \begin{array}{l} M_{\mathrm{rc}ki}=M_{rk1i}\cos(k\alpha_{t})-M_{rk2i}\sin(k\alpha_{t})\\ M_{\mathrm{rs}ki}=M_{rk1i}\sin(k\alpha_{t})+M_{rk2i}\cos(k\alpha_{t})\\ M_{\alpha cki}=M_{\alpha k1i}\cos(k\alpha_{t})-M_{\alpha k2i}\sin(k\alpha_{t})\\ M_{\alpha ski}=M_{\alpha k1i}\sin(k\alpha_{t})+M_{\alpha k2i}\cos(k\alpha_{t}) \end{array}\right.$$

where

(26) $$\left\{ \begin{array}{l} M_{rk1i}=(S_{1k1i}+S_{2k1i})B_{r}\alpha_{p}/\mu_{0}\\ M_{rk2i}=(S_{1k2i}+S_{2k2i})B_{r}\alpha_{p}/\mu_{0}\\ M_{\alpha k1i}=(-S_{1k2i}+S_{2k2i})B_{r}\alpha_{p}/\mu_{0}\\ M_{\alpha k2i}=(S_{1k1i}-S_{2k1i})B_{r}\alpha_{p}/\mu_{0} \end{array}\right.$$

(27) $$S_{1k1i}={\left(-1\right)}^{i}\frac{1}{2(k+1)\alpha_{p}\pi }\{\sin[\frac{(k+1)\alpha_{p}\pi +2ik\pi }{2p}]-\sin[\frac{(k+1)(1-2D)\alpha_{p}\pi +2ik\pi }{2p}]\}$$

(28) $$S_{2k1i}=\left\{ \begin{array}{ll} {\left(-1\right)}^{i}\frac{1}{2(k-1)\alpha_{p}\pi }\{\sin[\frac{(k-1)\alpha_{p}\pi +2ik\pi }{2p}]-\sin[\frac{(k-1)(1-2D)\alpha_{p}\pi +2ik\pi }{2p}]\},\; & k\neq 1\\ \frac{\alpha_{p}\pi D}{2p}\cos\frac{i\pi }{p}-\frac{1}{4}\cos\frac{i\pi }{p}[\sin(\frac{\alpha_{p}\pi }{p}+\frac{2\pi }{p}i)-\sin(\frac{\alpha_{p}(1-2D)\pi +2\pi i}{p})]- & \\ \frac{1}{4}\sin\frac{i\pi }{p}[\cos(\frac{\alpha_{p}\pi +2\pi i}{p})-\cos(\frac{\alpha_{p}(1-2D)\pi +2\pi i}{p})]-S_{1k1i}, & k=1 \end{array}\right.$$

(29) $$S_{1k2i}={\left(-1\right)}^{i}\frac{1}{2(k+1)\alpha_{p}\pi }\{-\cos[\frac{(k+1)\alpha_{p}\pi +2ik\pi }{2p}]+\cos[\frac{(k+1)(1-2D)\alpha_{p}\pi +2ik\pi }{2p}]\}$$

(30) $$S_{2k2i}=\left\{ \begin{array}{ll} {\left(-1\right)}^{i}\frac{1}{2(k-1)\alpha_{p}\pi }\{-\cos[\frac{(k-1)\alpha_{p}\pi +2ik\pi }{2p}]+\cos[\frac{(k-1)(1-2D)\alpha_{p}\pi +2ik\pi }{2p}]\}, & k\neq 1\\ \frac{\alpha_{p}\pi D}{2p}\sin\frac{i\pi }{p}-\frac{1}{4}\sin\frac{i\pi }{p}[\sin(\frac{\alpha_{p}\pi }{p}+\frac{2\pi }{p}i)-\sin(\frac{\alpha_{p}(1-2D)\pi +2\pi i}{p})]-\\ \frac{1}{4}\cos\frac{i\pi }{p}[\cos(\frac{\alpha_{p}\pi +2\pi i}{p})-\cos(\frac{\alpha_{p}(1-2D)\pi +2\pi i}{p}]-S_{1k2i}, & k=1 \end{array}\right.$$

The radial and tangential component of magnetization of healthy PMs are

(31) $$\left\{ \begin{array}{c} M_{r0}=\sum_{k}M_{rk0}\cos(k\alpha -k\alpha_{t})=\sum_{k}\left(M_{\mathrm{rc}k0}\cos k\alpha +M_{\mathrm{rs}k0}\sin k\alpha \right)\\ M_{\alpha 0}=\sum_{k}M_{\alpha k0}\cos(k\alpha -k\alpha_{t})=\sum_{k}\left(M_{\alpha ck0}\cos k\alpha +M_{\alpha sk0}\sin k\alpha \right) \end{array}\right.$$

(32) $$\{\begin{array}{c} M_{\mathrm{rc}k0}=M_{rk0}\cos(k\alpha_{t})\\ M_{\mathrm{rs}k0}=M_{rk0}\sin(k\alpha_{t})\\ M_{\alpha ck0}=-M_{\alpha k0}\sin(k\alpha_{t})\\ M_{\alpha sk0}=M_{\alpha k0}\cos(k\alpha_{t}) \end{array}$$

where

(33) $$M_{rk0}=\{\begin{array}{ll} (S_{1k}+S_{2k})B_{r}\alpha_{p}/\mu_{0}, & k/p=1,3,5\ldots \\ 0, & k/p\neq 1,3,5\ldots \end{array}$$

(34) $$M_{\alpha k0}=\{\begin{array}{ll} (S_{1k}-S_{2k})B_{r}\alpha_{p}/\mu_{0}, & k/p=1,3,5\ldots \\ 0, & k/p\neq 1,3,5\ldots \end{array}$$

(35) $$S_{1k}=\frac{\sin\left[(k+1)\alpha_{p}\pi /2p\right]}{(k+1)\alpha_{p}\pi /2p}$$

(36) $$S_{2k}=\{\begin{array}{ll} \frac{\sin\left[(k-1)\alpha_{p}\pi /2p\right]}{(k-1)\alpha_{p}\pi /2p}, & k\neq 1\\ 1, & k=1 \end{array}$$

According to (23) and (31), (22) can be transformed into

(37) $$\left\{ \begin{array}{c} M_{\mathrm{rD}}=-\sum_{i}\frac{a_{0ri}}{2}+\sum_{k}(M_{\mathrm{rc}k}\cos k\alpha +M_{\mathrm{rs}k}\sin k\alpha )\\ M_{\alpha D}=-\sum_{i}\frac{a_{0\alpha i}}{2}+\sum_{k}(M_{\alpha ck}\cos k\alpha +M_{\alpha sk}\sin k\alpha ) \end{array}\right.$$

(38) $$\{\begin{array}{c} M_{\mathrm{rc}k}=M_{\mathrm{rc}k0}-\sum_{i}M_{\mathrm{rc}ki}\\ M_{\mathrm{rs}k}=M_{\mathrm{rs}k0}-\sum_{i}M_{\mathrm{rs}ki}\\ M_{\alpha ck}=M_{\alpha ck0}-\sum_{i}M_{\alpha cki}\\ M_{\alpha sk}=M_{\alpha sk0}-\sum_{i}M_{\alpha ski} \end{array}$$

### 2.3. Motor Performance Calculation

The above analytical model is applicable to all PM motors with surface-mounted fan-shaped PMs, and the operation performances of the motor are calculated as follows.

The vector potential in the air gap (Region 2) and slot opening (Region 3) satisfies the Laplace equation.

(39) $$\frac{\partial^{2}A_{z}}{\partial r^{2}}+\frac{1}{r}\frac{\partial A_{z}}{\partial r}+\frac{1}{r^{2}}\frac{\partial^{2}A_{z}}{\partial \alpha^{2}}=0$$

The vector potential in the slot (Region 4) satisfies the Poisson equation.

(40) $$\frac{\partial^{2}A_{z}}{\partial r^{2}}+\frac{1}{r}\frac{\partial A_{z}}{\partial r}+\frac{1}{r^{2}}\frac{\partial^{2}A_{z}}{\partial \alpha^{2}}=-\mu_{0}J$$

The vector potential in the magnet (Region 1) satisfies the Poisson equation.

(41) $$\frac{\partial^{2}A_{z}}{\partial r^{2}}+\frac{1}{r}\frac{\partial A_{z}}{\partial r}+\frac{1}{r^{2}}\frac{\partial^{2}A_{z}}{\partial \alpha^{2}}=-\frac{\mu_{0}}{r}(M_{\alpha }-\frac{\partial M_{r}}{\partial \alpha })$$

where J is the current density of the slot. r and α represent the radial and tangential positions, respectively. $M_{r}$ and $M_{\alpha }$ are the radial and tangential components of magnet magnetization, respectively.

The boundary condition between each subdomain is the continuity of the vector potential and tangential magnetic field intensity at the interface of different subdomains, and one boundary with the rotor back iron has a condition that the tangential magnetic field intensity is zero.

(42) $$\{\begin{array}{c} \begin{array}{l} {H_{\alpha 1}|}_{r=R_{r}}=0\\ {A_{z1}|}_{r=R_{m}}={A_{z2}|}_{r=R_{m}} \end{array}\\ {H_{\alpha 1}|}_{r=R_{m}}={H_{\alpha 2}|}_{r=R_{m}}\\ {A_{z2}|}_{r=R_{s}}={A_{z3}|}_{r=R_{s}}\\ {H_{\alpha 2}|}_{r=R_{s}}={H_{\alpha 3}|}_{r=R_{s}}\\ {A_{z3}|}_{r=R_{t}}={A_{z4}|}_{r=R_{t}}\\ {H_{\alpha 3}|}_{r=R_{t}}={H_{\alpha 4}|}_{r=R_{t}} \end{array}$$

By solving the above equations, the expression of the vector potential in each subdomain can be obtained, to calculate the operation performances such as flux density, flux linkage, and back-EMF.

The radial and tangential components of flux density in the air gap can be given by

(43) $$\left\{ \begin{array}{c} B_{r2}=\frac{1}{r}\frac{\partial A_{z2}}{\partial \alpha }\\ B_{\alpha 2}=-\frac{\partial A_{z2}}{\partial r} \end{array}\right.$$

The flux linkage can be calculated from the vector potential

(44) $$\psi_{x}=\sum_{j}L_{z}\frac{N_{c}}{aS_{aj}}\iint_{S_{bj}}A_{z4}rdrd\alpha$$

where x=A,B,C denotes phases A,B,C of the stator winding, respectively. $L_{z}$ is the axial length of motor. $N_{c}$ is the number of turns per coil. a is the parallel branches per phase. $S_{aj}$ is the area of the jth slot; $S_{bj}$ is the area occupied by the winding in the jth slot.

The back-EMF of each phase can be calculated by

(45) $$E_{x}=-\frac{d\psi_{x}}{dt}$$

The output torque can be calculated by

(46) $$T=\frac{L_{z}r^{2}}{\mu_{0}}\int_{0}^{2\pi }B_{r2}B_{\alpha 2}d\alpha$$

## 3. Validation and Analysis

**Figure 8:**
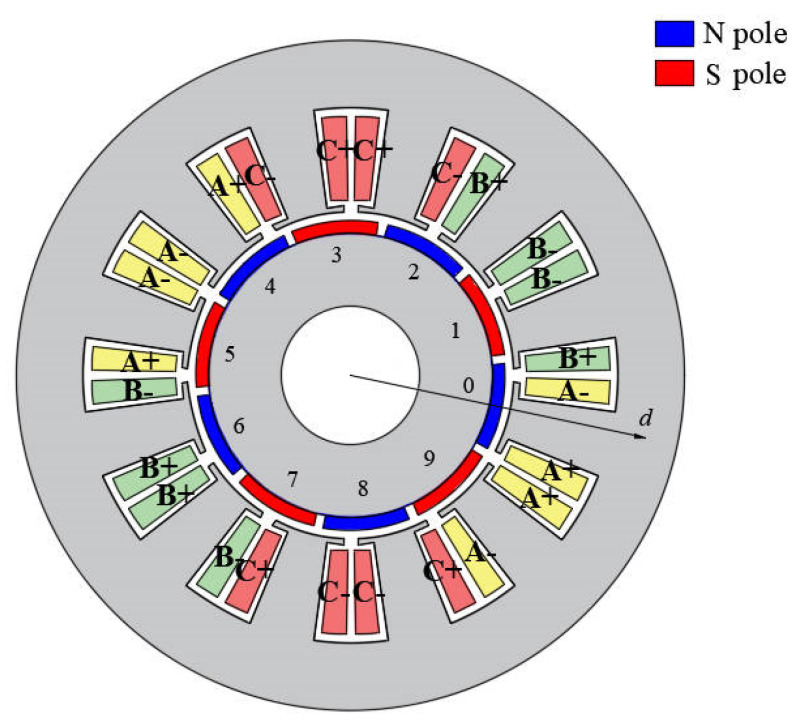
Structure of 10p/12s PM motor.

To verify the correctness of the proposed analytical model for PM motors with radially and parallel magnetized PMs, a FE model is made of a 10-pole 12-slot motor, as shown in Figure 8. PMs are assumed to have uniform demagnetization or partial demagnetization, and the results of the applied analytical model are compared with the FEA results. In the case of uniform demagnetization, the demagnetization degree of each magnet is 30% (D = 0.3), and in the case of partial demagnetization, the demagnetization degree of two adjacent PMs (numbered i 1 and 2 in Figure 8) is 30% (D = 0.3). The main parameters of the motor are shown in Table 1.

### 3.1. Performance Analysis of Motor with Radially Magnetized PMs

**Figure 9:**
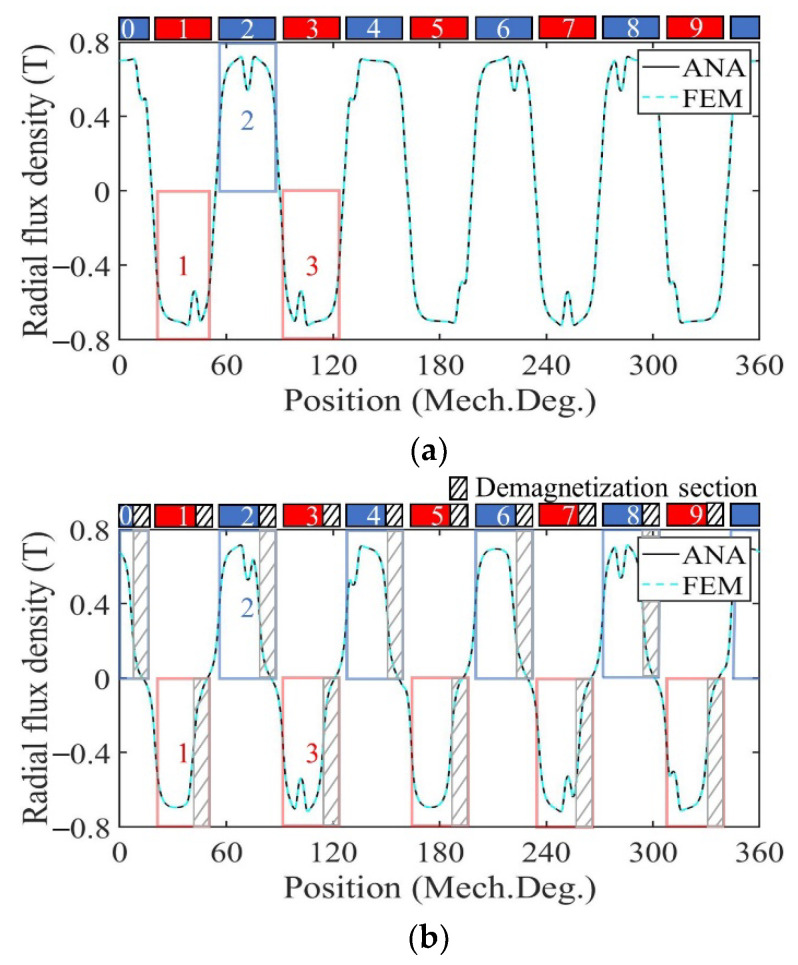

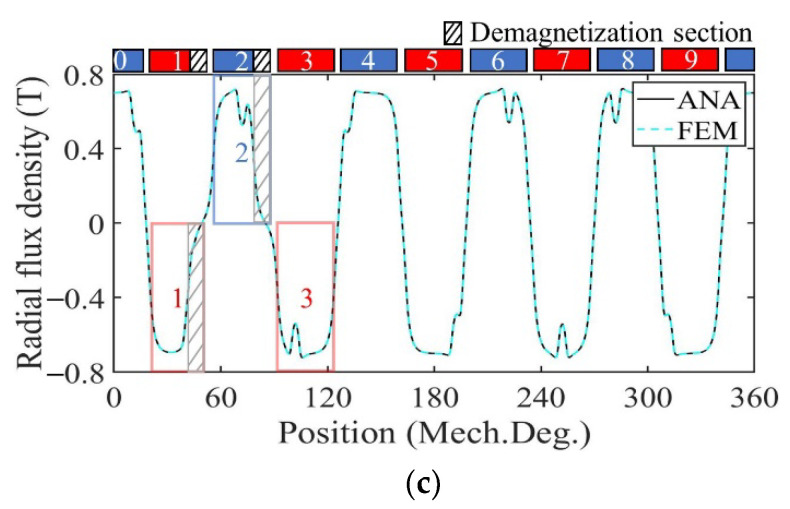
Radial air gap flux density comparison. (**a**) Healthy; (**b**) uniform demagnetization; (**c**) partial demagnetization.

Figure 9 shows the radial air gap flux density at no-load calculated by the analytical model and FE model in healthy, uniformly, and partially demagnetized states of the motor with a radially magnetized PM. For a demagnetized PM, the magnetization in the demagnetized region decreases to 0, and the amplitude of the no-load radial air gap flux density waveform decreases. In the case of uniform demagnetization, the flux density waveform of the shaded part in each PM in Figure 9b is significantly decreased compared with that of Figure 9a in the healthy state. In the case of partial demagnetization, the flux density waveform of the region numbered 1 and 2 in Figure 9c is basically consistent with that of Figure 9b, and the remaining areas are basically consistent with those of Figure 9a such as the region numbered 3. This is consistent with the theoretical analysis.

**Figure 10:**
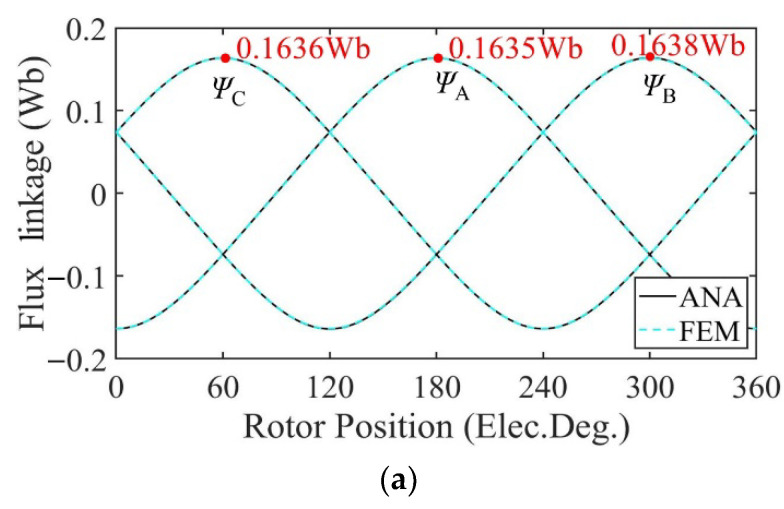

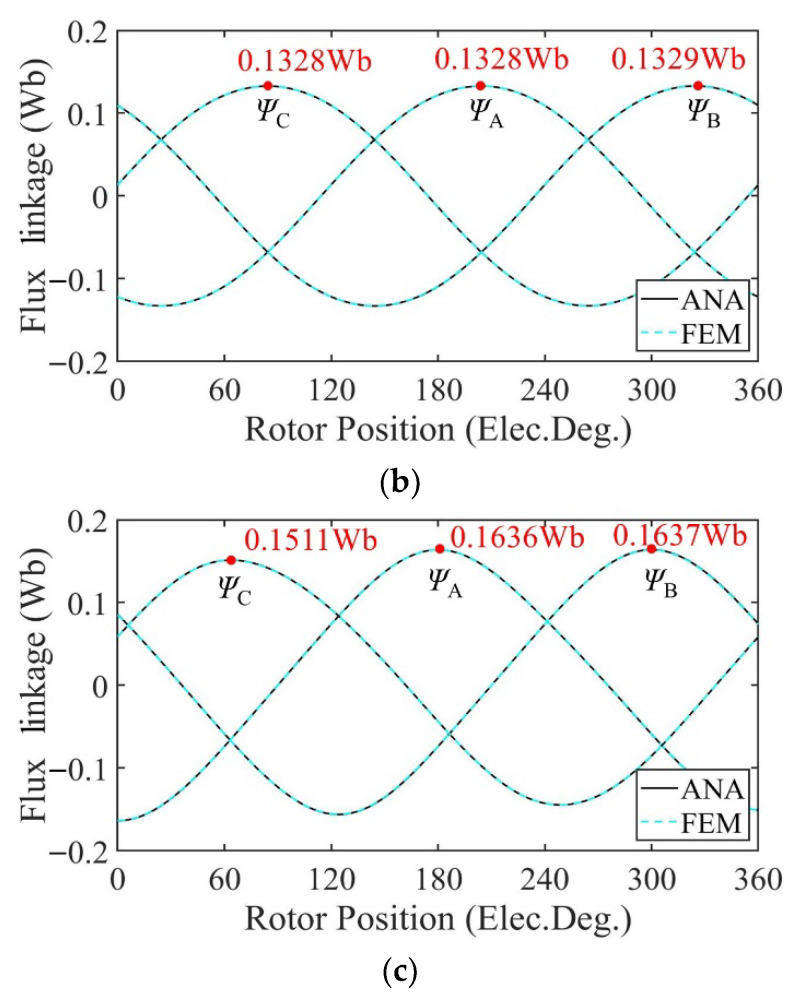
Flux linkage comparison. (**a**) Healthy; (**b**) uniform demagnetization; (**c**) partial demagnetization.

**Figure 11:**
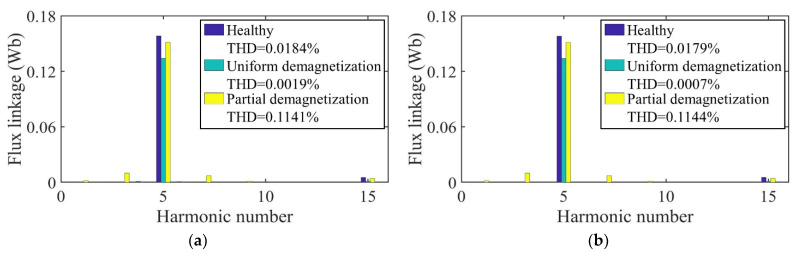
Harmonics in the flux-linkage relative to fundamental wave at fs = 166.67 Hz. (**a**) Analytical model; (**b**) FE model.

Figure 10 shows the no-load flux linkage of the motor in three cases calculated by the analytical model and the FE model at a speed of 2000 rpm. Because the distribution of the magnetic field generated by the PMs changes after demagnetization, the flux and flux linkage of the stator windings change accordingly. In the case of uniform demagnetization, the demagnetization degree of each PM is the same, and the magnetic field inside the motor remains evenly distributed. The amplitude of the three-phase no-load flux linkage under uniform demagnetization decreases by about 19% compared with the healthy condition. Under the circumstance of partial demagnetization, the magnetic field near the healthy magnets almost remains the same, while the magnetic field generated by the demagnetized PMs changes, resulting in an uneven distribution in the magnetic field and the introduction of harmonics into the three-phase no-load flux linkage. In Figure 10, the amplitude of the C-phase no-load flux linkage under partial demagnetization decreases by about 7.6% compared with the healthy condition. Taking one mechanical period as an example, the harmonic analysis of the no-load flux linkage of winding A under three conditions is carried out. The harmonic spectrum is shown in Figure 11. The no-load flux linkage of the motor under the healthy condition and uniform demagnetization condition is mainly composed of the fundamental wave and 3rd harmonic. In addition, the amplitude of the fundamental wave and 3rd harmonic under the uniform demagnetization condition decreases by about 15.4% and 79.6%, respectively, compared with the healthy condition. In the case of partial demagnetization, the amplitude of the fundamental wave decreases by about 4.4% compared with the healthy condition, and the total harmonic distortion rate (THD) is about 6 times the healthy condition.

**Figure 12:**
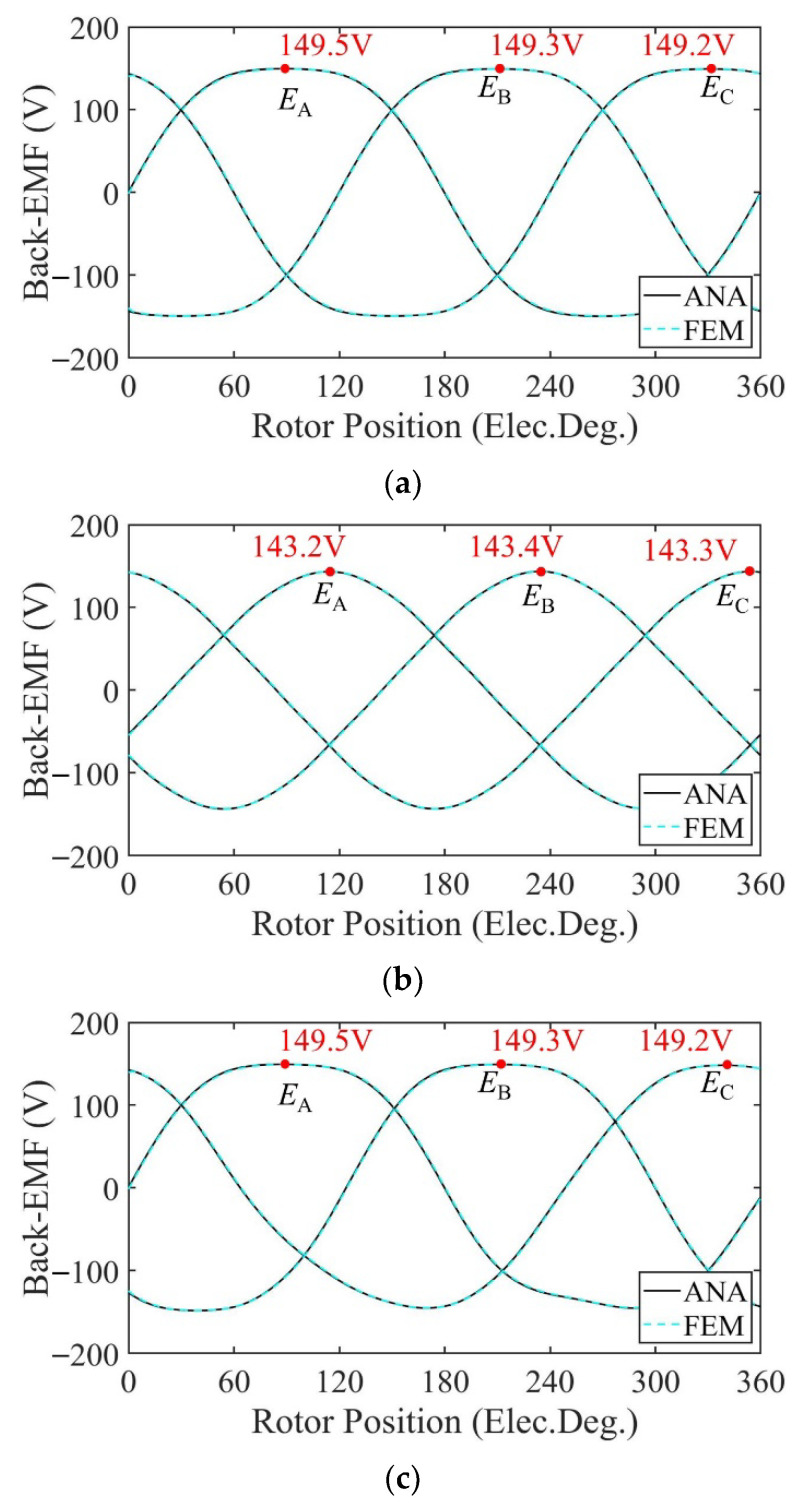
Back-EMF comparison. (**a**) Healthy; (**b**) uniform demagnetization; (**c**) partial demagnetization.

Figure 12 shows the no-load back-EMF of the motor calculated by the two models at a speed of 2000 rpm. Because the distribution of the magnetic field generated by the PMs changes after demagnetization, the no-load back-EMF induced by flux variation in the windings changes accordingly. In the case of uniform demagnetization, the magnetic field still maintains an even distribution. The amplitude of the no-load back-EMF under uniform demagnetization decreases by about 4% compared with the healthy condition. Under the circumstance of partial demagnetization, the magnetic field generated by the PMs becomes uneven, introducing harmonics into the three-phase no-load back-EMF. The waveforms of the three-phase no-load back-EMF under partial demagnetization have obvious distortion.

**Figure 13:**
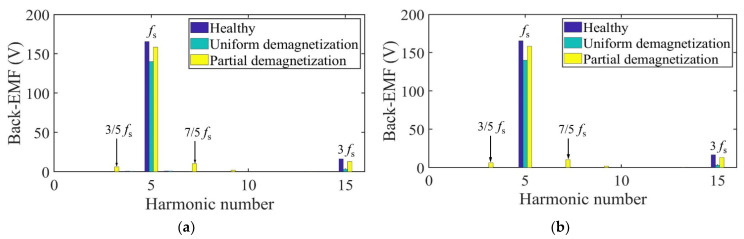
Harmonics in the back-EMF relative to fundamental wave at fs = 166.67 Hz. (**a**) Analytical model; (**b**) FE model.

Taking one mechanical period as an example, the harmonic analysis of the no-load back-EMF of winding A under three conditions is carried out. The harmonic spectrum is shown in Figure 13. The no-load back-EMF of the motor under the healthy condition and uniform demagnetization condition is mainly composed of the fundamental wave and 3rd harmonic. In addition, the amplitude of the fundamental wave and 3rd harmonic under uniform the demagnetization condition decreases by about 15.4% and 79.6%, respectively, compared with the healthy condition. In the case of partial demagnetization, in addition to the fundamental wave and the 3rd harmonics, the fault characteristic harmonics will also appear in the no-load back-EMF [30], resulting in the back-EMF distortion. The frequencies of the fault characteristic harmonics are

(47) $$f_{\mathrm{de}}=(k_{d}/p)f_{s}\;k_{d}=1,2,3\ldots$$

where $f_{\mathrm{de}}$ is the fault characteristic frequencies in the spectrum, and $f_{s}$ is the fundamental electrical frequency of the back-EMF.

Due to the pole-slot combination of the motor (*p* = 5, s = 12), the fault signatures at the 2/5th harmonic frequency and multiples of it (even multiples of the mechanical frequency) in phase A are suppressed [31].

Table 2 shows the normalized content of each harmonic calculated by the fast Fourier transform (FFT) of the no-load back-EMF of winding A, obtained by the analytical model and FE model of the motor under three conditions. The error between the analytical results and FEA results can be obtained by

(48) ε=|ANA−FEM|

where ANA represents the harmonic percentage share calculated by the analytical model. FEM represents the harmonic percentage share calculated by the FE model.

As can be seen from Table 2, due to the influence of partial demagnetization, fractional harmonics such as 1/5th, 3/5th, and 7/5th appear in the no-load back-EMF spectrum, which is consistent with the theoretical analysis. The maximum relative error of each harmonic is 0.203%. The analytical and FEA data have good correspondence with each other.

**Figure 14:**
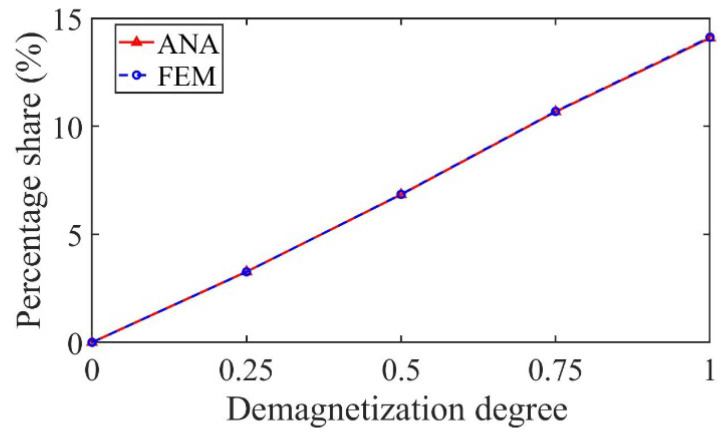
Variation in the percentage share of the harmonic with demagnetization degree.

As shown in Figure 14, taking the 3/5th harmonic as an example, the percentage share of the harmonic increases with the increase in the demagnetization degree, which can be used as the fault characteristic harmonic to judge the partial demagnetization degree.

**Figure 15:**
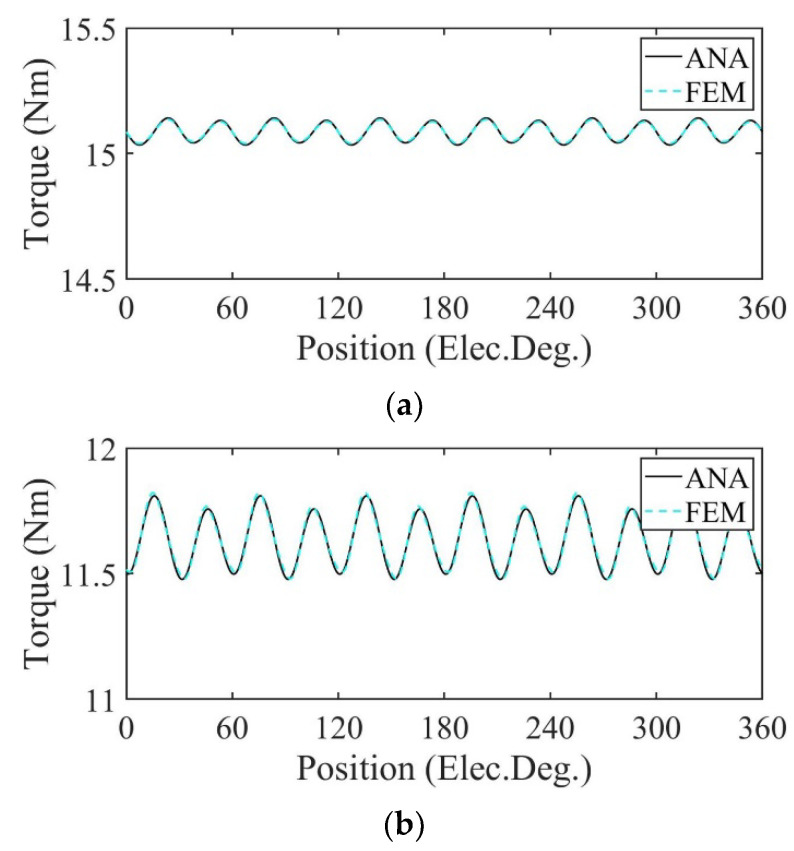

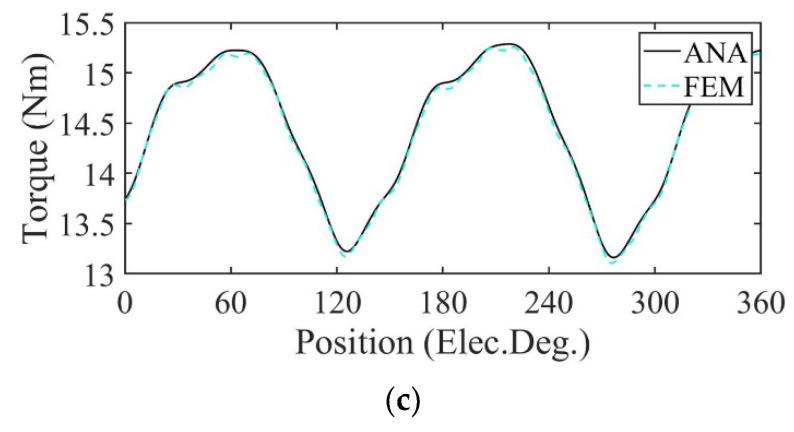
Output torque comparison. (**a**) Healthy; (**b**) uniform demagnetization; (**c**) partial demagnetization.

**Figure 16:**
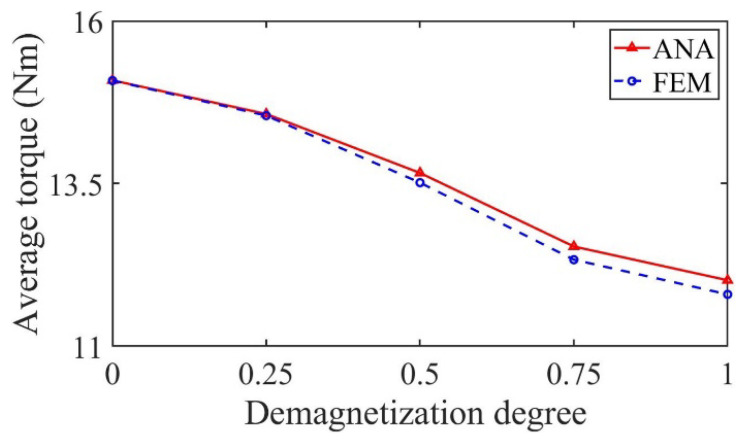
Variation in the average torque with demagnetization degree.

In Figure 15 and Figure 16, the motors are operated at rated load. Figure 15 shows the output torque calculated by the analytical model and FE model of the motor. Under the uniform demagnetization condition and partial demagnetization condition, the average output torque decreases by about 23% and 4% compared with the healthy condition, respectively. Because of partial demagnetization, rotor symmetry changes. Hence, the period of the output torque waveform increases. Meanwhile, the increase in harmonics leads to the increase in torque ripple.

Figure 16 shows the variation in average output torque with demagnetization degree under partial demagnetization. It can be seen from the figure that the average output torque decreases with the increase in demagnetization degree, so it can be used as one of the fault signatures to judge the degree of partial demagnetization.

Table 3 shows the comparison of the root mean square (RMS) and errors of flux density, flux linkage, back-EMF, and output torque of the motor under three conditions. The relative error δ is calculated by

(49) $$\delta =\left|\frac{Y_{\mathrm{ANA}}-Y_{\mathrm{FEM}}}{Y_{\mathrm{FEM}}}\right|\times 100\%$$

where $Y_{\mathrm{ANA}}$ represents the RMS of results calculated by the analytical model. $Y_{\mathrm{FEM}}$ represents the RMS of results calculated by the FE model.

As can be seen from Table 3, the maximum relative errors of the radial air gap flux density, flux linkage, back-EMF, and output torque are 0.0595%, 0.0914%, 0.1646%, and 0.2425%, respectively. The analytical and FEA data have a good correspondence with each other.

### 3.2. Performance Analysis of Motor with Parallel Magnetized PMs

**Figure 17:**
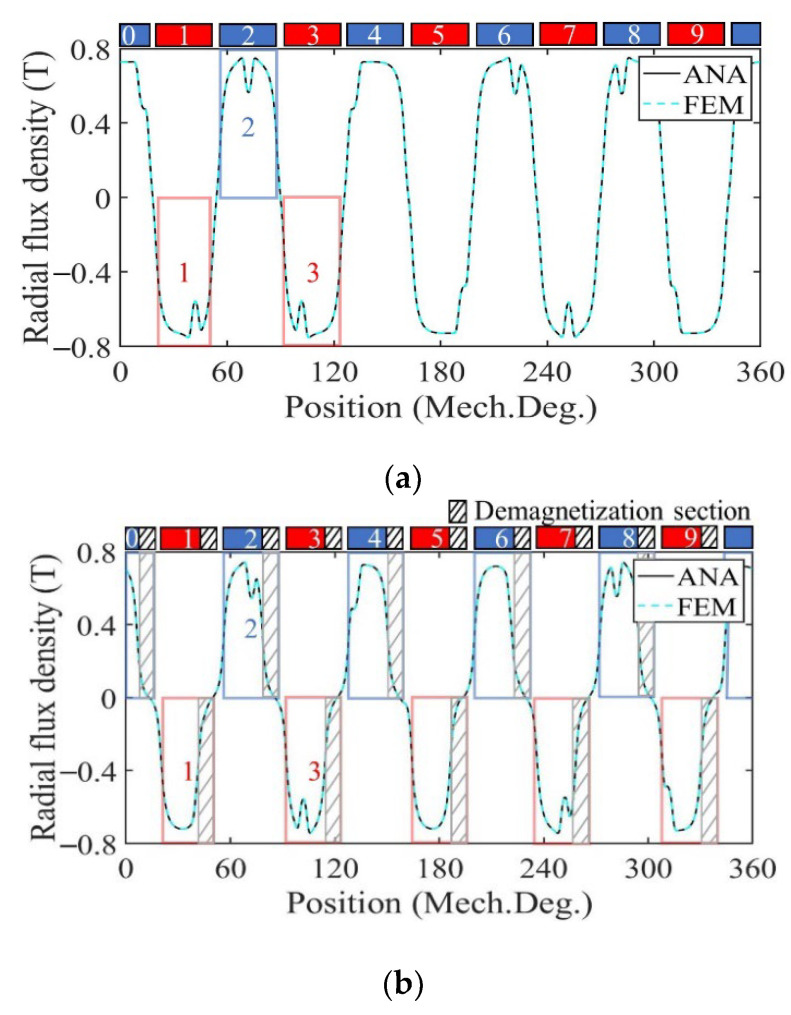

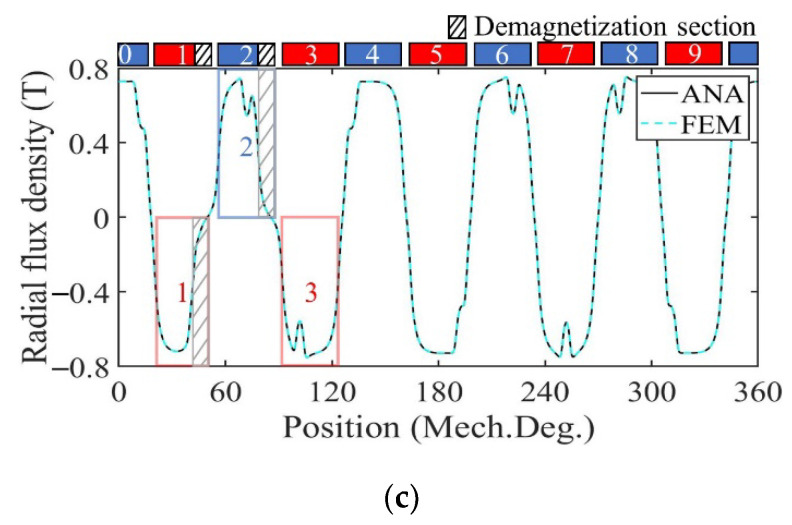
Radial air gap flux density comparison. (**a**) Healthy; (**b**) uniform demagnetization; (**c**) partial demagnetization.

Figure 17 shows the radial air gap flux density at no-load calculated by the analytical model and FE model in healthy, uniform, and partial demagnetization states of the motor with parallel magnetized PM. For demagnetized PM, the magnetization in the demagnetized region decreases to 0, and the amplitude of the no-load radial air gap flux density waveform decreases. In the case of uniform demagnetization, the flux density waveform of the shaded part in each PM in Figure 17b is significantly decreased compared with that of Figure 17a in the healthy state. In the case of partial demagnetization, the flux density waveform of the region numbered 1 and 2 in Figure 17c is basically consistent with that of Figure 17b, and the remaining areas are basically consistent with those of Figure 17a such as the region numbered 3. This is consistent with the theoretical analysis.

**Figure 18:**
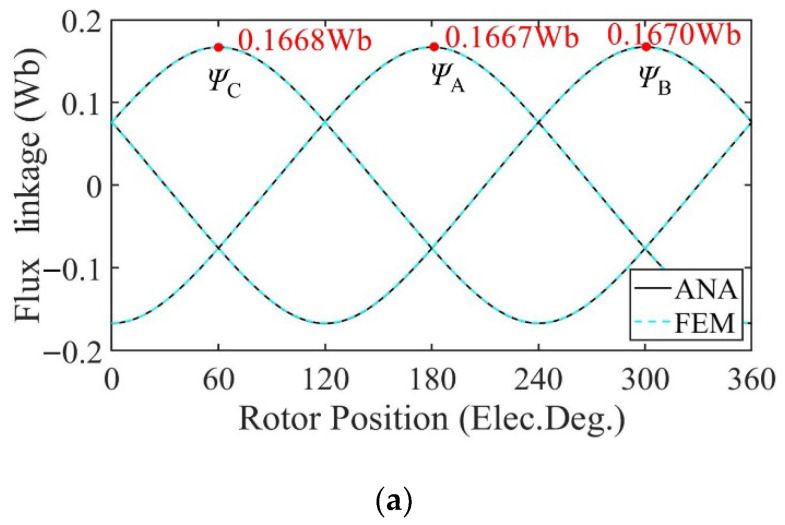

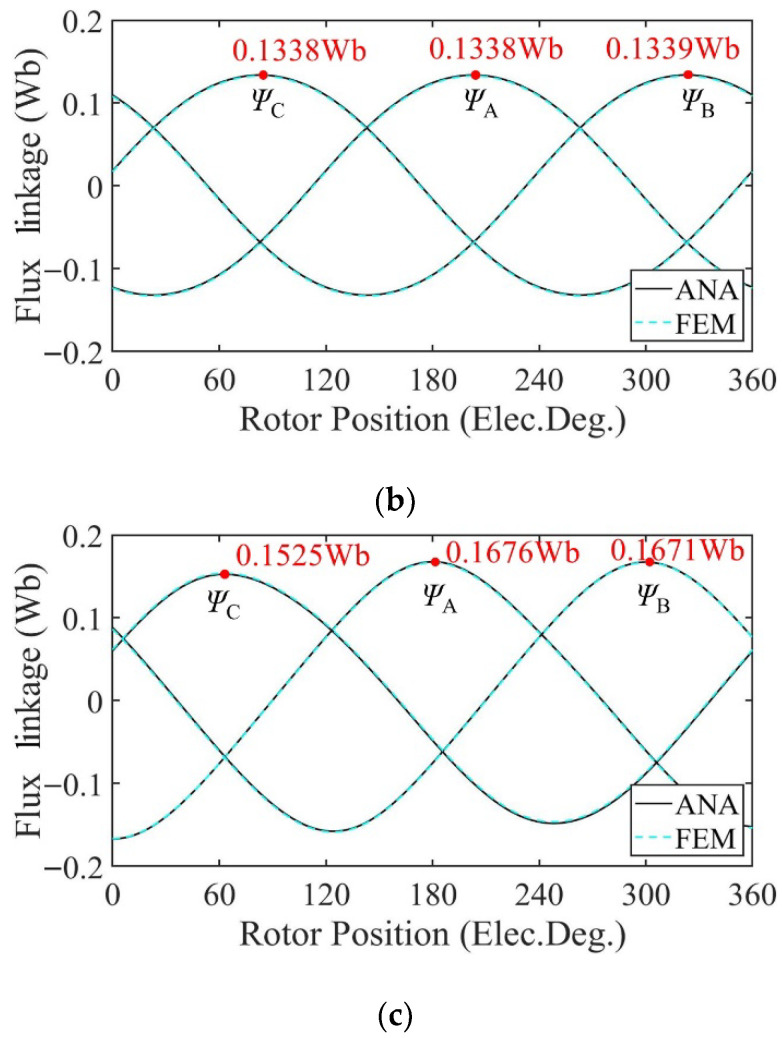
Flux linkage comparison. (**a**) Healthy; (**b**) uniform demagnetization; (**c**) partial demagnetization.

**Figure 19:**
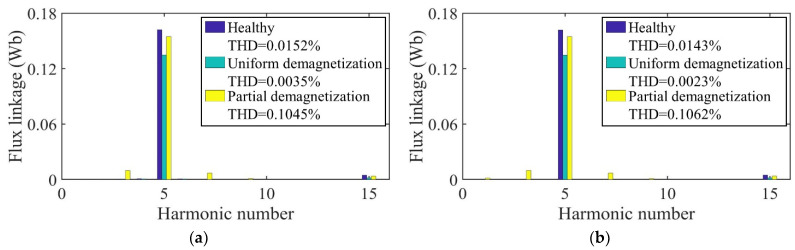
Harmonics in the flux-linkage relative to fundamental wave at fs = 166.67 Hz. (**a**) Analytical model; (**b**) FE model.

Figure 18 shows the no-load flux linkage of the motor under three conditions calculated by the analytical model and the FE model at a speed of 2000 rpm. Because the distribution of the magnetic field generated by the PMs changes after demagnetization, the flux and flux linkage of the stator windings change accordingly. In the case of uniform demagnetization, the demagnetization degree of each PM is the same, and the magnetic field inside the motor remains evenly distributed. The amplitude of the three-phase no-load flux linkage under uniform demagnetization decreases by about 19.8% compared with the healthy condition. Under the circumstance of partial demagnetization, the magnetic field near the healthy magnets almost remains the same, while the magnetic field generated by the demagnetized PMs changes, resulting in an uneven distribution in the magnetic field and the introduction of harmonics into the three-phase no-load flux linkage. In Figure 18, the amplitude of the C-phase no-load flux linkage under partial demagnetization decreases by about 7.6% compared with the healthy condition. Taking one mechanical period as an example, the harmonic analysis of the no-load flux linkage of winding A under three conditions is carried out. The harmonic spectrum is shown in Figure 19. The no-load flux linkage of the motor under the healthy condition and uniform demagnetization condition is mainly composed of the fundamental wave and 3rd harmonic. In addition, the amplitude of the fundamental wave and 3rd harmonic under the uniform demagnetization condition decreases by about 16.7% and 61.8%, respectively, compared with the healthy condition. In the case of partial demagnetization, the amplitude of the fundamental wave decreases by about 4.4% compared with the healthy condition, and the total harmonic distortion rate (THD) is about 7 times the healthy condition.

**Figure 20:**
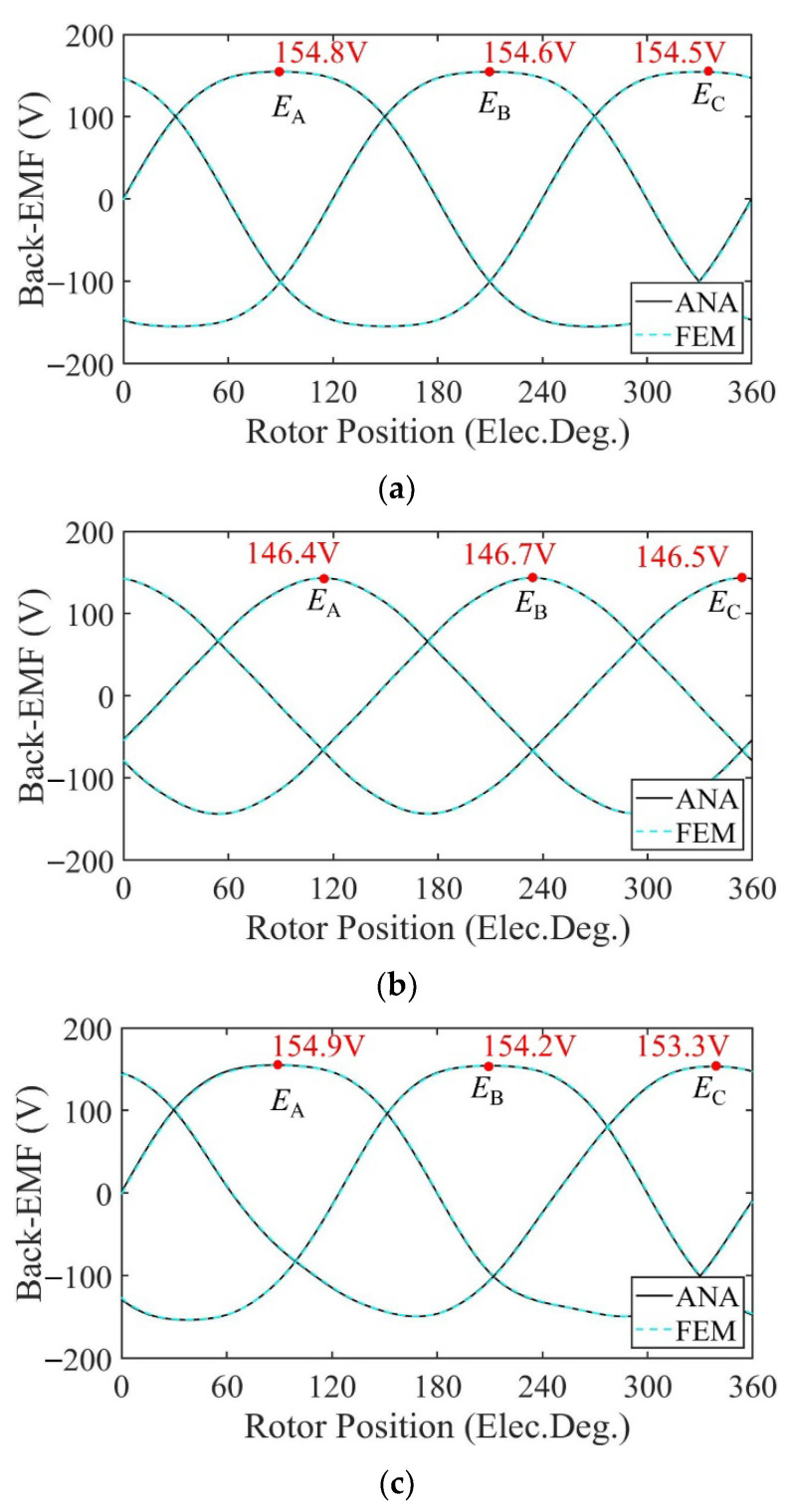
Back-EMF comparison. (**a**) Healthy; (**b**) uniform demagnetization; (**c**) partial demagnetization.

Figure 20 shows the no-load back-EMF of the motor calculated by the two models at a speed of 2000 rpm. Because the distribution of the magnetic field generated by the PMs changes after demagnetization, the no-load back-EMF induced by flux variation in the windings changes accordingly. In the case of uniform demagnetization, the magnetic field still maintains an even distribution. The amplitude of the no-load back-EMF under uniform demagnetization decreases by about 5% compared with the healthy condition. Under the circumstance of partial demagnetization, the magnetic field generated by the PMs becomes uneven, introducing harmonics into the three-phase no-load back-EMF. The waveforms of the three-phase no-load back-EMF under partial demagnetization have obvious distortion.

**Figure 21:**
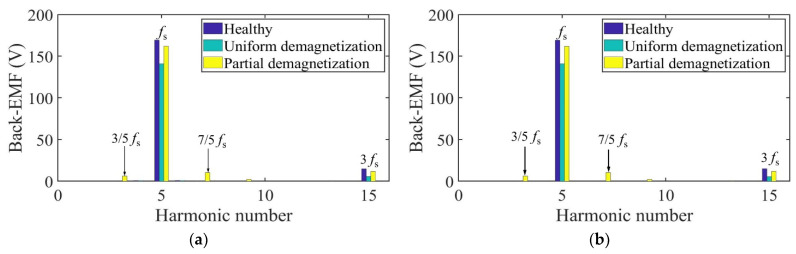
Harmonics in the back-EMF relative to fundamental wave at fs = 166.67 Hz. (**a**) Analytical model; (**b**) FE model.

Taking one mechanical period as an example, the harmonic analysis of the no-load back-EMF of winding A under three conditions is carried out. The harmonic spectrum is shown in Figure 21. The no-load back-EMF of the motor under the healthy condition and uniform demagnetization condition is mainly composed of the fundamental wave and 3rd harmonic. In addition, the amplitude of the fundamental wave and 3rd harmonic under uniform demagnetization condition decreases by about 16.9% and 61.8%, respectively, compared with the healthy condition.

Table 4 shows the normalized content of each harmonic calculated by the FFT of the no-load back-EMF of winding A, obtained by the analytical model and FE model of the motor under three conditions.

As can be seen from Table 4, due to the influence of partial demagnetization, fractional harmonics such as 1/5th, 3/5th, and 7/5th appear in the no-load back-EMF spectrum, which is consistent with the theoretical analysis. The maximum relative error of each harmonic is 0.199%. The analytical and FEA data have good correspondence with each other.

**Figure 22:**
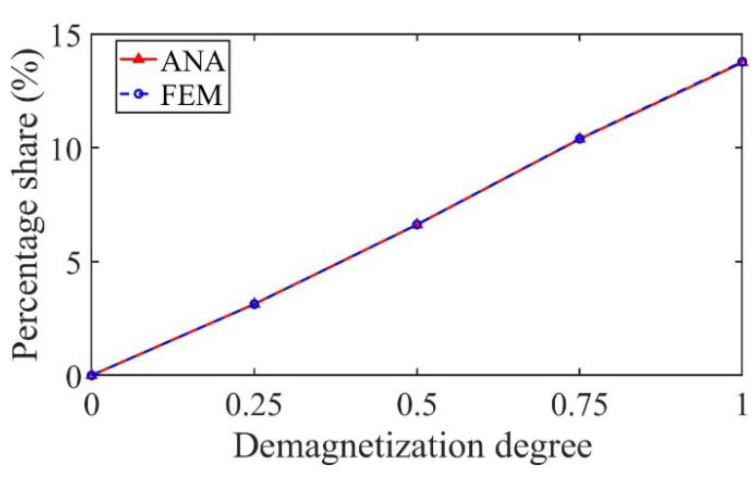
Variation in the percentage share of the harmonic with demagnetization degree.

As shown in Figure 22, taking the 3/5th harmonic as an example, the percentage share of the harmonic increases with the increase in the demagnetization degree, which can be used as the fault characteristic harmonic to judge the partial demagnetization degree.

**Figure 23:**
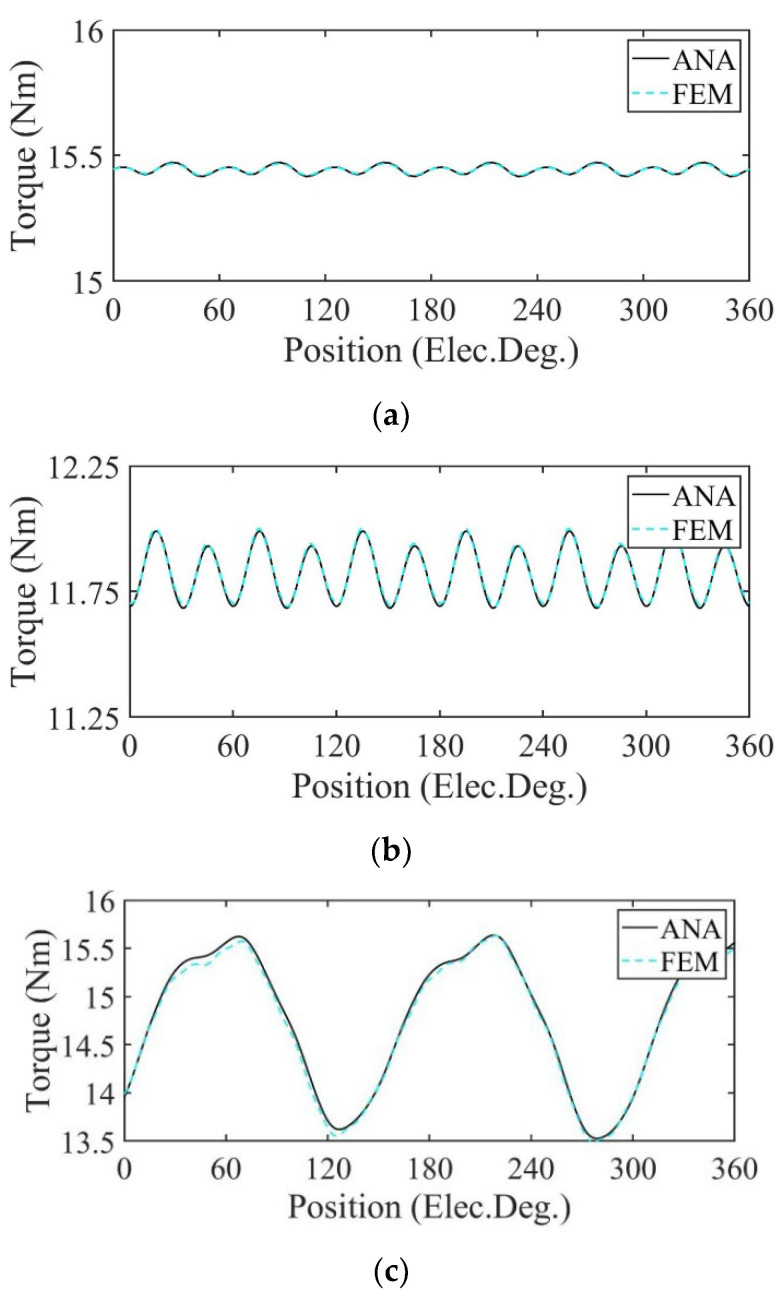
Output torque comparison. (**a**) Healthy; (**b**) uniform demagnetization; (**c**) partial demagnetization.

**Figure 24:**
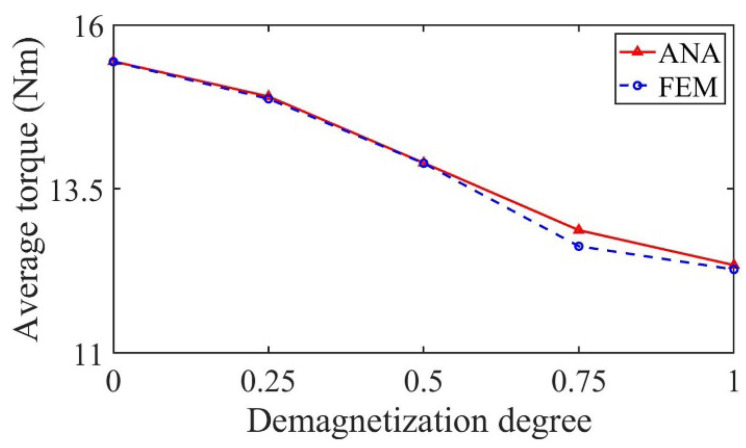
Variation in the average torque with demagnetization degree.

In Figure 23 and Figure 24, the motors are operated at rated load. Figure 23 shows the output torque calculated by the analytical model and FE model of the motor. Under the uniform demagnetization condition and partial demagnetization condition, the average output torque decreases by about 23% and 4% compared with the healthy condition, respectively. Because of partial demagnetization, rotor symmetry changes. Hence, the period of the output torque waveform increases. Meanwhile, the increase in harmonics leads to the increase in torque ripple.

Figure 24 shows the variation in average output torque with demagnetization degree under partial demagnetization. It can be seen from the figure that the average output torque decreases with the increase in demagnetization degree, so it can be used as one of the fault signatures to judge the degree of partial demagnetization.

Table 5 shows the comparison of the RMS and errors of flux density, flux linkage, back-EMF, and output torque of the motor under three conditions.

As can be seen from Table 5, the maximum relative errors of radial air gap flux density, flux linkage, back-EMF, and output torque of the two models are 0.0503%, 0.0893%, 0.2168%, and 0.2630%, respectively. The analytical and FEA data have a good correspondence with each other.

For motors at rated load, the comparison of calculation time required for an electrical period (100 time-steps per period) using the analytical model and FE model is shown in Table 6. As can be seen from the table, the calculation time of the analytical model is about 1/20 of the FE model. The analytical method has a faster calculation speed, which is beneficial to the early demagnetization fault diagnosis.

## 4. Conclusions

In this paper, a novel strategy for the demagnetization fault modeling of PM motors is proposed. The change in the Fourier coefficients in the Fourier expansion of the magnetization waveform of PMs is introduced to represent the uniformly and the partially demagnetized PMs with either radial or parallel magnetization. This modeling method based on the demagnetization region is also applicable to PMs with different magnetization directions, shapes, and demagnetization types. This model is used to analyze different demagnetization types, and the following conclusions are obtained:

(1) In the case of uniform demagnetization, the flux, back-EMF, and average output torque of the motor decrease.

(2) In the case of partial demagnetization, fractional harmonics appear in the back-EMF spectrum, and its amplitude can be used to judge the severity of partial demagnetization.

(3) The average torque can also be used to estimate the severity of partial demagnetization.

This demagnetization fault modeling method solves the problem of modeling the non-uniform magnetic field distribution caused by PM demagnetization. Compared with other methods, it has the advantages of clear physical relationships between various parameters, fast calculation speed, and high accuracy. Using this analytical model, the theoretical operation performance of different structures and operating conditions of the motor with demagnetization faults can be obtained quickly, which provides a reference for further real-time fault diagnosis, prediction, and maintenance planning.

## Figures and Tables

**Table 1:** Main parameters of PM motor.

Parameter	Symbol	Value	Unit
Pole pair	p	5	- -
Slot/tooth number	s	12	- -
Rated speed	$n_{N}$	2000	rpm
Rated current	$I_{N}$	9	A
Axial length	$L_{z}$	125	mm
Radius of slot bottom	$R_{\mathrm{sb}}$	64	mm
Radius of slot top	$R_{t}$	40.5	mm
Inner radius of stator	$R_{s}$	39	mm
Outer radius of rotor	$R_{r}$	34	mm
Airgap length	g	2	mm
Slot opening width	$b_{\mathrm{oa}}$	π/36	rad
Slot width	$b_{\mathrm{sa}}$	4π/45	rad
Magnet thickness	$h_{m}$	3	mm
Residual flux density of PM	$B_{r}$	1.23	T
Relative permeability of PM	$\mu_{r}$	1.03	- -
Pole-arc to pole-pitch ratio	$\alpha_{p}$	0.9	- -
Number of turns per coil	$N_{c}$	54	- -
Number of Parallel Branches	a	2	- -

**Table 2:** Comparison of harmonic components of back-EMF and error.

Motor Status	Harmonic Frequency (Hz)	$k_{d}$	ANA (%)	FEM (%)	ε (%)
Healthy	166.7 (fs)	5	100	100	0.000
	500	15	9.875	10.078	0.203
Uniform demagnetization	166.7 (fs)	5	100	100	0.000
	500	15	2.376	2.231	0.145
Partial demagnetization	33.3	1	0.209	0.212	0.003
	100	3	3.953	3.953	0.000
	166.7 (fs)	5	100	100	0.000
	233.3	7	6.596	6.571	0.025
	300	9	1.257	1.248	0.009
	500	15	8.214	8.332	0.118
	566.7	17	2.934	2.922	0.012
	633.3	19	0.935	0.936	0.001

**Table 3:** Comparison of RMS and error for PM motor with radially magnetized PMs.

		Healthy	Uniform Demagnetization	Partial Demagnetization
$B_{r2}$ (T)	ANA	0.6067	0.5037	0.5874
	FEM	0.6065	0.5040	0.5873
	δ (%)	0.0330	0.0595	0.0170
$\psi_{A}$ (Wb)	ANA	0.1120	0.0947	0.1095
	FEM	0.1119	0.0947	0.1094
	δ (%)	0.0894	0.0007	0.0914
$E_{A}$ (V)	ANA	117.6990	99.1365	115.0123
	FEM	117.5056	99.0559	114.8373
	δ (%)	0.1646	0.0814	0.1524
T (Nm)	ANA	15.0868	11.6360	14.4665
	FEM	15.0832	11.6390	14.4315
	δ (%)	0.0239	0.0258	0.2425

**Table 4:** Comparison of harmonic components of back-EMF and error.

Motor Status	Harmonic Frequency (Hz)	$k_{d}$	ANA (%)	FEM (%)	ε (%)
Healthy	166.7 (fs)	5	100	100	0.000
	500	15	8.776	8.866	0.090
Uniform demagnetization	166.7 (fs)	5	100	100	0.000
	500	15	4.029	3.905	0.124
Partial demagnetization	33.3	1	0	0.002	0.002
	100	3	0.004	0.203	0.199
	166.7 (fs)	5	3.792	3.792	0.000
	233.3	7	100	100	0.000
	300	9	6.348	6.338	0.010
	500	15	1.218	1.208	0.010
	566.7	17	7.190	7.289	0.099
	633.3	19	2.922	2.906	0.016

**Table 5:** Comparison of RMS and error for PM motor with parallel magnetized PMs.

		Healthy	Uniform Demagnetization	Partial Demagnetization
$B_{r2}$ (T)	ANA	0.6167	0.5082	0.5966
	FEM	0.6164	0.5083	0.5963
	δ (%)	0.0487	0.0197	0.0503
$\psi_{A}$ (Wb)	ANA	0.1146	0.0952	0.1121
	FEM	0.1145	0.0952	0.1120
	δ (%)	0.0873	0.0219	0.0893
$E_{A}$ (V)	ANA	120.3556	99.7624	117.6695
	FEM	120.1492	99.6588	117.4150
	δ (%)	0.1718	0.1040	0.2168
T (Nm)	ANA	15.4431	11.8246	14.7890
	FEM	15.4383	11.8290	14.7502
	δ (%)	0.0311	0.0372	0.2630

**Table 6:** Computation time comparison at rated load (Unit: s).

Motor Status	Radial Magnetization		Parallel Magnetization	
	ANA	FEM	ANA	FEM
Healthy	33	791	34	826
Uniform demagnetization	37	661	37	676
Partial demagnetization	44	794	45	805

## Data Availability

Not applicable.
